# Kidney Injury Causes Accumulation of Renal Sodium That Modulates Renal Lymphatic Dynamics

**DOI:** 10.3390/ijms23031428

**Published:** 2022-01-27

**Authors:** Jing Liu, Elaine L. Shelton, Rachelle Crescenzi, Daniel C. Colvin, Annet Kirabo, Jianyong Zhong, Eric J. Delpire, Hai-Chun Yang, Valentina Kon

**Affiliations:** 1 Department of Nephrology, Tongji University School of Medicine, Shanghai 200070, China; liujing961226@163.com; 2 Department of Pediatrics, Vanderbilt University Medical Center, Nashville, TN 37232, USA; 3 Department of Pharmacology, Vanderbilt University Medical Center, Nashville, TN 37232, USA; elaine.l.shelton@vumc.org; 4 Department of Radiology, Vanderbilt University Medical Center, Nashville, TN 37232, USA; rachelle.crescenzi@vumc.org (R.C.); daniel.colvin@vumc.org (D.C.C.); 5 Department of Medicine, Division of Clinal Pharmacology and Department of Molecular Physiology and Biophysics, Vanderbilt University Medical Center, Nashville, TN 37232, USA; annet.kirabo@vanderbilt.edu (A.K.); jianyong.zhong@vumc.org (J.Z.); 6 Department of Pathology, Microbiology and Immunology, Vanderbilt University Medical Center, Nashville, TN 37232, USA; eric.delpire@vanderbilt.edu; 7 Department of Anesthesiology, Vanderbilt University Medical Center, Nashville, TN 37232, USA

**Keywords:** kidney, lymphatics, sodium, NKCC1 transporter

## Abstract

Lymphatic vessels are highly responsive to changes in the interstitial environment. Previously, we showed renal lymphatics express the Na-K-2Cl cotransporter. Since interstitial sodium retention is a hallmark of proteinuric injury, we examined whether renal sodium affects NKCC1 expression and the dynamic pumping function of renal lymphatic vessels. Puromycin aminonucleoside (PAN)-injected rats served as a model of proteinuric kidney injury. Sodium ^23^Na/^1^H-MRI was used to measure renal sodium and water content in live animals. Renal lymph, which reflects the interstitial composition, was collected, and the sodium analyzed. The contractile dynamics of isolated renal lymphatic vessels were studied in a perfusion chamber. Cultured lymphatic endothelial cells (LECs) were used to assess direct sodium effects on NKCC1. MRI showed elevation in renal sodium and water in PAN. In addition, renal lymph contained higher sodium, although the plasma sodium showed no difference between PAN and controls. High sodium decreased contractility of renal collecting lymphatic vessels. In LECs, high sodium reduced phosphorylated NKCC1 and SPAK, an upstream activating kinase of NKCC1, and eNOS, a downstream effector of lymphatic contractility. The NKCC1 inhibitor furosemide showed a weaker effect on ejection fraction in isolated renal lymphatics of PAN vs controls. High sodium within the renal interstitium following proteinuric injury is associated with impaired renal lymphatic pumping that may, in part, involve the SPAK-NKCC1-eNOS pathway, which may contribute to sodium retention and reduce lymphatic responsiveness to furosemide. We propose that this lymphatic vessel dysfunction is a novel mechanism of impaired interstitial clearance and edema in proteinuric kidney disease.

## 1. Introduction

Sodium retention is a well-documented consequence of many pathophysiological conditions, especially kidney disease, which is clinically recognized as an accumulation of edema [1]. Previous studies found sodium retention in skin and muscle is connected to blood pressure regulation involving lymphatic remodeling [2,3,4]. Recent research indicates that sodium, along with water, accumulates systemically, including in the lung, liver, muscle, and myocardium [5,6]. While kidneys have a central role in regulating sodium homeostasis, few studies have quantified kidney sodium or water content, including in edema-forming conditions. Such studies have been primarily limited by a lack of methodology for sodium quantification in vivo. Recent developments in noninvasive sodium imaging by ^23^Na-MRI provide an attractive tool for quantifying kidney sodium content in vivo. Moreover, although kidney disease is regularly accompanied by lymphatic vessel hyperplasia [7,8,9,10,11,12,13,14], whether disease-induced lymphangiogenesis is accompanied by disrupted renal lymphatic vessel dynamics is unknown. Lymphatics are important because unlike blood flow, which relies on the heart as a central pump, lymph flow is propelled by forces in the surrounding tissues and by active rhythmic contractions intrinsic to the lymphatic vessels themselves. These intrinsic mechanisms constitute a major force in lymphatic flow and are exquisitely sensitive to the microenvironment, for example, hydraulic pressure, shear stress, local tissue temperature, and sodium [15]. A recent study provides evidence that lymphangiogenesis accompanying arthritis in TNF-transgenic mice reflects intrinsic dysfunction in popliteal lymphatic vessels that is linked to NOS-dependent as well as independent impairment in lymphatic vessel dynamics that may drive arthritic damage of the joint [16]. Whether intrarenal sodium modulates the renal lymphatic contractions has not been reported.

Lymphatic vessel contractility is driven by action potentials that trigger Ca++ influx generated by ion channels and transporters. We recently showed the Na-K-2Cl cotransporter NKCC1, but not NKCC2, is expressed in renal lymphatic vessels [17]. While NKCC2 is best known for its actions on tubular epithelial cells responsible for the maintenance of sodium homeostasis, NKCC1 is increasingly recognized as a modulator of various unanticipated biological functions, including regulation of vascular tone [18]. Indeed, inhibition of NKCC1 and its activating kinases has become a novel antihypertensive strategy involving direct (non-diuretic) vascular dilation. However, in contrast to blood vessels, little is known about NKCC transporter expression, activity, or function in the lymphatic vascular network and how the microenvironment or disease alters these parameters. This is particularly relevant since the first line of intervention in the treatment of edema and underlying interstitial clearance impairment is NKCC inhibition by furosemide. 

Here we assessed whether kidney injury affects renal sodium content, how a high- sodium environment alters the pumping dynamics of renal collecting lymphatic vessels, and the role of NKCC1 in this response.

## 2. Results

### 2.1. Proteinuric Kidney Injury Increased Renal Sodium

**Figure 1:**
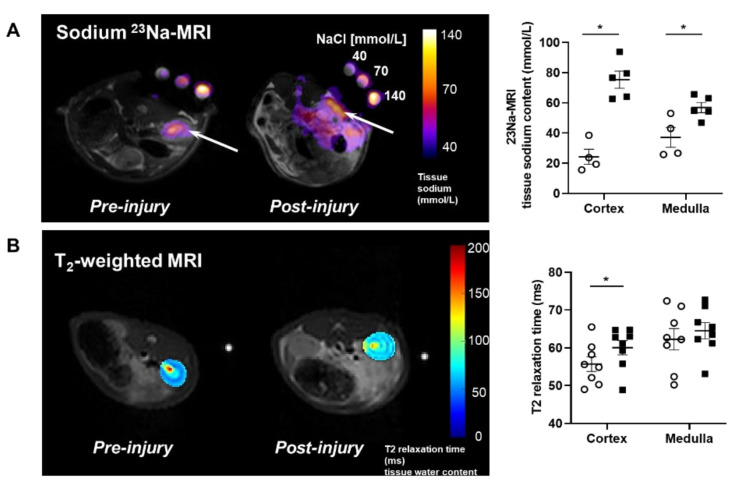
Proteinuric kidney injury increased renal sodium and water content. (**A**) Representative sodium ^23^Na-MRI in uninjured control (Cont) (open circles) and puromycin (PAN)-injured rats (closed squares). The graph shows mean tissue sodium content localized in the cortex and medulla of the kidneys (arrows). (**B**) Representative T_2_-weighted MRI images and quantitative T_2_-relaxation time measurements indicative of renal cortical and medullary water content in Cont and PAN-injured rats. Results are expressed as mean ± SEM. n = 5 to 8 rats per group analyzed by unpaired *t* test. * *p* < 0.05.

**Figure 2:**
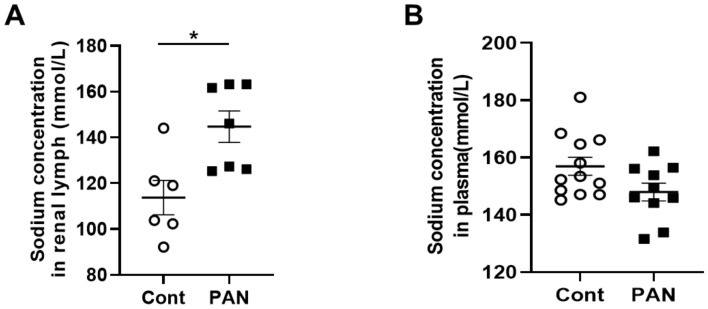
Proteinuric kidney injury increased sodium concentration in renal lymph. (**A**) Analysis of renal lymph showed higher sodium concentration in PAN-injured (closed squares) vs control rats (open circles). (**B**) Analysis of plasma showed no difference in sodium concentration between PAN-injured vs control rats. Results are expressed as mean ± SEM for 6 to 8 rats per group analyzed by unpaired *t* test. * *p* < 0.05.

MRI analysis revealed that puromycin aminonucleoside nephropathy (PAN) injury leads to increased renal sodium content. Both the cortex and medulla in PAN-injured rats had significantly higher sodium than in control rats (Figure 1A). The renal cortex of PAN rats also showed increased water content compared with controls. Although a directionally similar trend occurred in the medulla of PAN-injured rats, the increase did not reach statistical significance (Figure 1B). In companion studies, we measured the sodium concentration in the lymph exiting the kidney, which is thought to reflect the composition within the renal interstitial compartment. These direct measurements revealed that sodium concentration in the renal lymph of PAN rats was significantly higher than in the lymph of control rats (Figure 2A). Sodium concentration in concurrently obtained serum samples was not different between PAN rats and controls (Figure 2B). These results indicate that, in addition to the well-documented proteinuria, hypoalbuminemia, and hyperlipidemia, PAN kidney injury leads to intrarenal sodium and water retention, especially in the renal cortex. Similar to dermal lymphatics of hypertensive animals, which transport excess sodium from the skin [4], our results show for the first time that renal lymphatics are a route for clearing excess sodium from the renal interstitium in the setting of proteinuric kidney injury.

### 2.2. High Sodium Environment Changed Contractility of Renal Lymphatic Vessels Involving the NKCC1 Transporter

**Figure 3:**
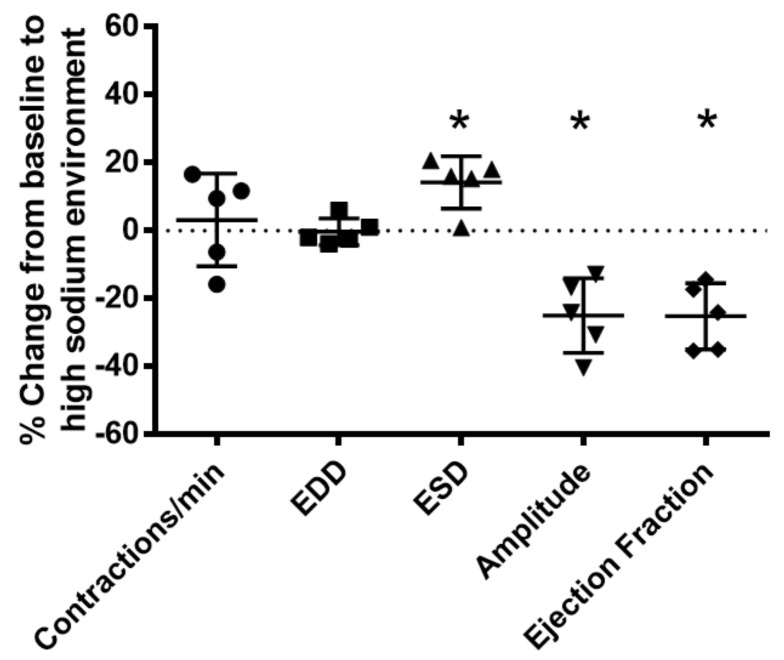
High salt environment altered renal collecting lymphatic vessel pumping function. Extra−renal afferent lymphatic vessels were subjected to a high−sodium buffer (185 mmol Na^+^ Krebs solution). A digital image capture system was used to measure the following vessel pumping parameters: frequency of spontaneous contractions, end diastolic lumen diameter (EDD), end systolic lumen diameter (ESD), contraction amplitude, and ejection fraction. Exposure to a high−sodium environment caused a significant increase in ESD, resulting in a significant decrease in amplitude and ejection fraction. Data points represent the percent change from measurements captured under baseline conditions (143 mmol Na^+^ Krebs solution) and are expressed as mean ± SEM. n = 5 individual vessels isolated from 5 rats. Significance was assessed by analyzing raw measurements using an unpaired *t* test. * *p* < 0.05.

To determine the effects of a high-sodium environment on renal lymphatic function, we measured the vasodynamics of renal afferent collecting lymphatic vessels. Similar to studies in afferent skin lymphatics [19], the contractility was assessed in renal afferent vessels isolated from normal rats exposed to normal buffer containing 143 mmol Na+ Krebs solution and compared with dynamics following exposure to Krebs solution containing a sodium concentration of 185 mmol. Compared with the physiologic buffer, a high-sodium environment had little effect on lymphatic contraction frequency. However, although high sodium caused only subtle changes in end diastolic diameter (EDD), a pronounced increase in end systolic diameter (ESD) contributed to reduced contraction amplitude and ejection fraction compared with the physiologic buffer (Figure 3).

**Figure 4:**
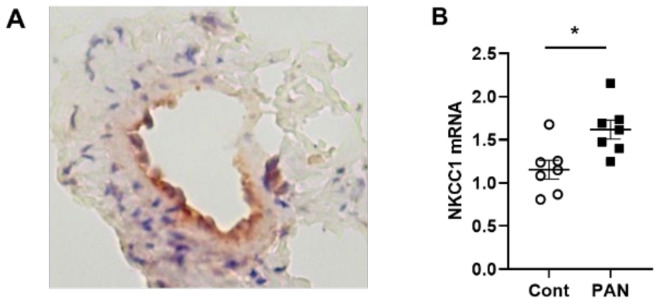
NKCC-1 transporter expression in renal lymphatic vessels and vessels with PAN proteinuric injury. (**A**) Immunostaining of afferent renal lymphatic vessels demonstrated NKCC1 transporter expression, particularly prominent in lymphatic endothelial cells. (**B**) NKCC1 mRNA levels in extra-renal lymphatic vessels were significantly greater in PAN-injured rats vs uninjured controls. Results are mean ± SEM for 7 rats per group analyzed by unpaired *t* test * *p* < 0.05.

**Figure 5:**
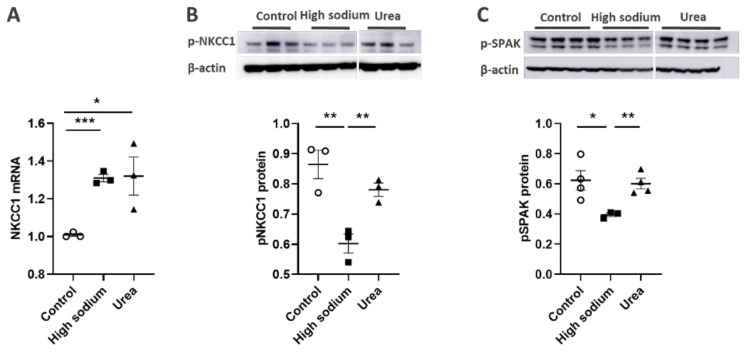
High Na^+^ environment regulated NKCC-1 signaling pathway in lymphatic endothelial cells (LECs). (**A**) Cultured LECs exposed to a high-sodium environment showed greater expression of NKCC1 mRNA, (**B**) while expression of phosphorated NKCC-1 protein decreased. (**C**) High-sodium environment decreased protein expression of SPAK, an upstream activating kinase of NKCC1. Experiments were performed independently 3 times using 3 wells per treatment and analyzed by ANOVA followed by Dunnett multiple comparisons. * *p* < 0.05, ** *p* < 0.01, *** *p* < 0.001.

**Figure 6:**
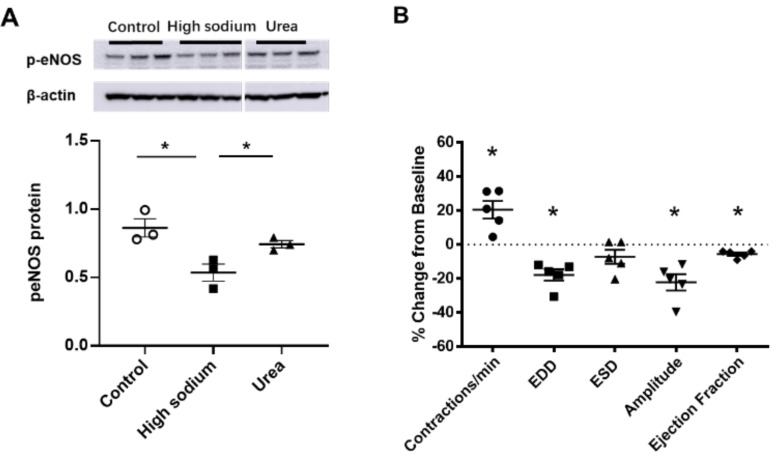
eNOS modulated lymphatic vessel function. (**A**) Cultured LECs exposed to high-sodium, but not high-osmolar environment showed reduced eNOS activity. (**B**) Isolated renal lymphatic vessels challenged with the eNOS inhibitor, L-NAME, exhibited increased contraction frequency and reduced EDD, amplitude, and ejection fraction. EDD, end diastolic diameter; ESD, end systolic diameter. Protein concentration results are expressed as mean ± SEM for 3 samples analyzed by ANOVA followed by Dunnett multiple comparison test. Vessel pumping parameters are expressed as the percent change from measurements captured under baseline conditions (143 mmol Na^+^ Krebs solution) and are expressed as mean ± SEM for 5 individual vessels isolated from 5 rats. Significance was assessed by analyzing raw measurements using an unpaired *t* test. * *p* < 0.05.

NKCC1 regulates blood vessel dynamics, and our previous study confirmed expression of NKCC1 in lymphatic endothelial cells. However, it is unknown whether NKCC1 has a role in modulating microenvironmental influences on lymphatic vessel dynamics. This is interesting, as lymphatic vessels were recently reported to regulate sodium homeostasis. Renal lymphangiogenesis in mice with kidney-specific overexpression of VEGF-D increased urinary sodium excretion and reduced systemic blood pressure in salt-loaded hypertensive mice but not normotensive basal conditions [20]. The mechanism was linked to downregulation of sodium transporters, namely, total NCC and ENaCα in tubular epithelial cells. NKCC1 expression and renal lymphatic function were not evaluated. Our immunohistochemical staining verified prominent expression of NKCC1 in afferent renal lymphatic vessels (Figure 4A). Moreover, NKCC1 gene expression was increased in vessels from PAN-injured rats compared with controls (Figure 4B). Also, LECs exposed to a high-sodium environment had elevated NKCC1 mRNA compared with cells maintained in media with physiological levels of sodium (Figure 5A). Similar upregulation in NCKK1 mRNA occurred in response to urea that is equimolar to high sodium exposure. Since NKCC activity is determined by phosphorylation, we assessed phosphorated-NKCC1 protein. Our results show that a high-sodium environment significantly reduced expression of phosphorated-NKCC1 protein while the hyperosmolar urea control did not (Figure 5A). Furthermore, as NKCC activity is linked to phosphorylation of WNK-SPAK/OSR1 signaling cascade, we also examined expression of this upstream kinase [21,22]. Our data show that a high-sodium environment also reduced phosphorylated SPAK compared with the baseline sodium control group (Figure 5C) [21]. Among the vasoactive factors, eNOS is a major endothelial-derived mechanism regulating lymphatic dynamics, which is regulated by activity of NKCC1 [23]. Exposing LECs to a high-sodium environment caused a significant reduction in eNOS activity as measured by the amount of phosphorylated eNOS protein (Figure 6A). In contrast, increased osmolarity with urea did not significantly alter the endothelial eNOS activity although the eNOS activity was significantly higher than in cells exposed to a high-sodium environment, echoing reduced p-eNOS levels shown in cardiac tissues of rats fed a high-salt diet [24]. To determine the consequences of reduced NO signaling on renal lymphatic vessel pumping dynamics, we exposed isolated vessels to L-NAME in order to inhibit eNOS activity. This caused a significant increase in contraction frequency, but reduced EDD, magnitude of contraction, and ejection fraction (Figure 6B).

### 2.3. Kidney Injury Diminished the Lymphatic Vascular Response to a High-Sodium Environment and NKCC Inhibition by Furosemide

**Figure 7:**
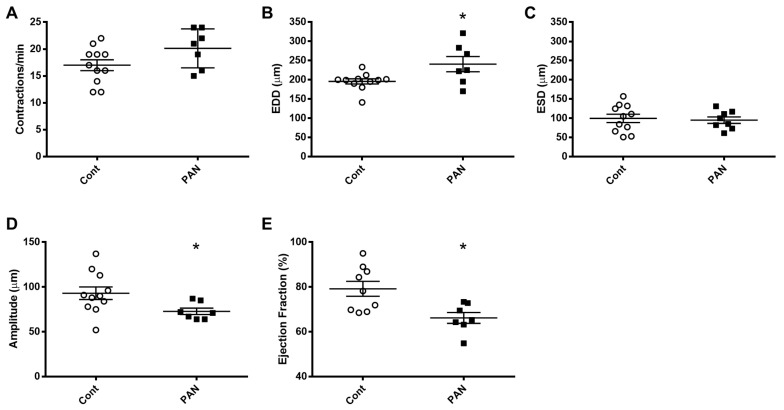
Kidney injury diminishes lymphatic vessel pumping efficiency. Vasodynamic parameters were measured in renal lymphatic vessels isolated from control and PAN-injured rats. Vessels from PAN-injured rats had significantly increased EDD (**B**), resulting in reduced contraction amplitude (**D**) and ejection fraction (**E**), while contraction frequency (**A**) and ESD (**C**) remained unchanged. Datapoints represent raw measurements from individual vessels isolated from 7 to 11 rats per group. Results are expressed as mean ± SEM analyzed by unpaired *t* test. * *p* < 0.05 EDD, end diastolic diameter; ESD, end systolic diameter.

**Figure 8:**
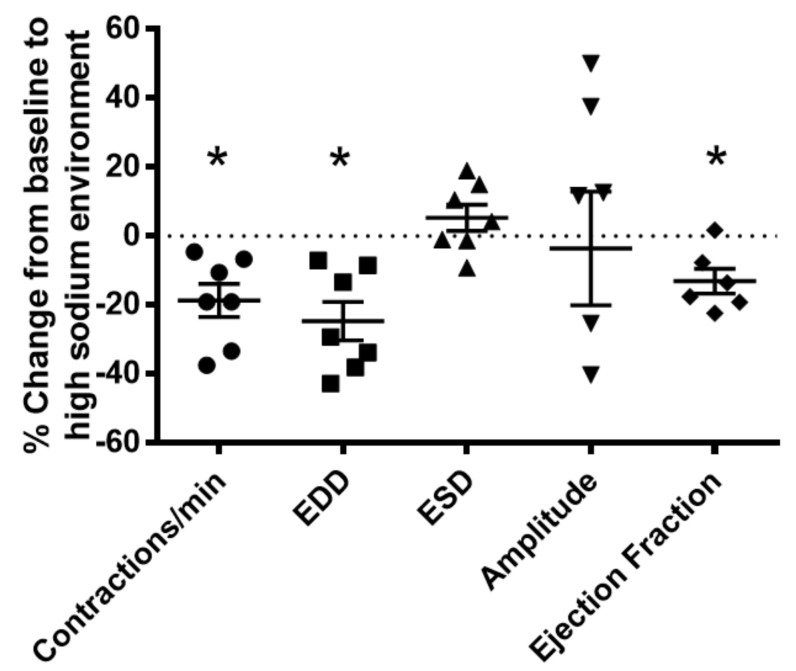
Vasodynamic response of PAN-injured lymphatic vessels exposed to a high-sodium environment. PAN-injured vessels exposed to high sodium had a significant decrease in the frequency of spontaneous contractions, EDD, and ejection compared with PAN-injured vessels in a normal sodium environment. Data points represent the percent change from measurements captured under normal sodium conditions and are expressed as mean ± SEM for 6 to 7 individual vessels isolated from 6 to 7 rats. Significance was assessed by analyzing raw measurements using an unpaired *t* test. * *p* < 0.05. EDD, end diastolic diameter; ESD, end systolic diameter.

To gain further insight into the effects of kidney injury on renal lymphatic physiology, we compared the pumping dynamics of control vessels with vessels from a PAN-injured rat in a normal sodium environment (Figure 7). PAN-injured vessels had a significant increase in EDD (Figure 7B), which contributed to a marked decrease in contraction amplitude (Figure 7D) and ejection fraction (Figure 7E) compared with control vessels. Next, to investigate how a high-sodium environment affects vessels in the setting of kidney injury, we compared the lymphatic dynamics in the vessels of PAN-injured rats before and after exposure to a high-sodium environment (Figure 8). PAN-injured lymphatic vessels had a distinct response to a high-sodium environment compared with the response of control vessels (Figure 3), exhibiting a decreased EDD, and a decreased ejection fraction. This suggests that a high-sodium environment and PAN injury both result in reduced ejection fraction, albeit by different mechanisms. 

**Figure 9:**
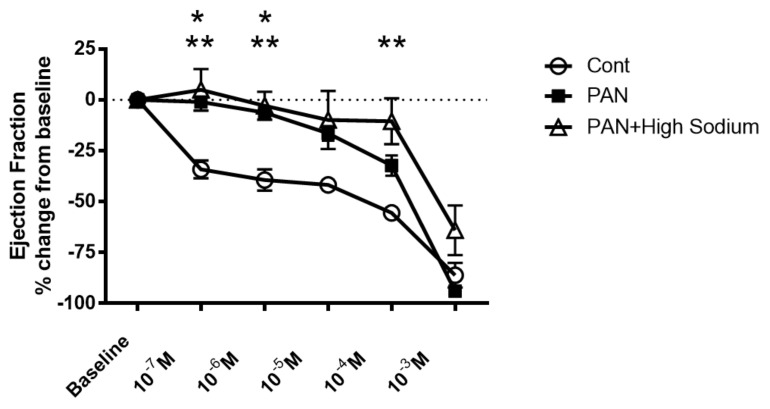
Kidney injury and exposure to elevated sodium blunt lymphatic vessel response to NKCC inhibition by furosemide. Renal lymphatic vessels isolated from PAN-injured rats and normal controls were subjected to increasing concentrations of the NKCC antagonist furosemide. Some PAN vessels were challenged with furosemide in a high-sodium environment. In control vessels, furosemide induces a robust decrease in ejection fraction. In contrast, PAN-injured vessels in normal and high-sodium environments are significantly less sensitive to furosemide. Data points represent the percent change from measurements captured at baseline conditions and are expressed as mean ± SEM for <6 individual vessels isolated from <6 rats. Significance (*p* < 0.05) was analyzed by ANOVA followed by Dunnett multiple comparisons. * PAN vessels compared with control vessels, ** PAN vessels in high sodium compared with control vessels.

Since injured lymphatic vessels appear to have weaker intrinsic compensatory responses to the high-sodium environment likely prevailing in a disease setting, we examined the effects of the NKCC1 inhibitor furosemide in PAN-injured and control vessels. Control vessels treated with furosemide had a pronounced concentration-dependent decrease in ejection fraction. In contrast, furosemide had more subtle effects on the ejection fraction of PAN-injured vessels, with PAN vessels being significantly less affected by furosemide at physiologically relevant doses (Figure 9). Interestingly, there was no statistical difference in the effects of furosemide on PAN vessels in a normal or high-sodium environment. These results indicate that analogous to a high-sodium environment and PAN-induced kidney injury, furosemide exerts directionally similar moderating effects on lymphatic dynamics that may affect renal interstitial clearance.

## 3. Discussion

A high-sodium environment is a critical modulator of lymphatic vessels. Although kidneys are central in Na+ homeostasis, little is understood about Na+ effects on renal lymphatics. The current studies provide new insights into regulation of renal lymphatic network by showing (1) proteinuric kidney injury increases renal Na+ by ^23^Na/1H MRI and direct sampling of renal lymphatic fluid shows elevated Na+ concentration while plasma Na+ is unchanged (2) high Na+ and furosemide inhibition of NKCC1 decrease lymphatic vessel contraction amplitude and ejection fraction in isolated renal lymphatic vessels, (3) a high Na+ environment decreases phosphorated-NKCC1, phosphorylated SPAK, an upstream kinase, and phosphorylated eNOS, a downstream vasoactive factor, and (4) a high Na+ environment together with renal injury contribute to a blunted lymphatic response in PAN-injured kidneys.

Noninvasive imaging by ^23^Na/1H MRI showed that proteinuric kidney injury leads to accumulation of sodium and water in the in vivo kidneys. This new observation reflects advances in multi-nuclear imaging technology that exploit endogenous ^23^Na, the second most abundant magnetic nuclei in living systems [25]. Imaging methods are advantageous for longitudinal measurement of tissue sodium before and after intervention, localization of tissue sodium in renal sub-compartments, and comparison of multi-modal data, strategies explored in this study. The findings of this study demonstrate ^23^Na-MRI quantification of renal sodium as a potential biomarker of renal disease involving lymphatic clearance dysfunction. Imaging results, supported by data, suggest that lymph exiting proteinuric kidneys has significantly higher sodium concertation than the renal lymph of normal, uninjured control rats. Sodium levels in the blood of these proteinuric animals were not different from normal rats. To date, there are only sparse data on the composition of renal lymph, especially in disease settings, although more than 50 years ago, two studies describing partial occlusion of the inferior vena cava model of right heart failure found increased renal lymphatic flow and sodium content [26,27]. More recently, sodium accumulation in the skin of salt-sensitive hypertensive rats was shown to be accompanied by increased sodium concentration in lymph collected from dermal lymphatic vessels, while no change in the circulating level of sodium was observed [4]. These findings reinforce the concept that lymph reflects the composition of the interstitial compartment of the draining organ. Our data make the original observation that kidney injury leads to renal sodium accumulation, although the study did not localize sodium to any specific interstitial compartment [1]. Sodium accumulation in the interstitium has been linked to the modulation of lymphatic vessels, especially lymphangiogenesis. This has been most extensively studied in the skin of hypertensive animals and humans and involves transcription factor tonicity-responsive enhancer protein (TonEBP)-induced macrophage secretion of vascular endothelial growth factor-C (VEGF-C) [4]. Although kidney injury causes renal lymphangiogenesis and modulates sodium reabsorption and excretion, there have been no studies on the possible effects of accumulating interstitial sodium on renal lymphatic function. We now show that direct exposure of renal lymphatic vessels to a high-sodium environment increases the frequency of contraction in the renal collecting lymphatic vessels and reduces the contraction amplitude, and, to a lesser extent, the ejection fraction (Figure 3). These results complement findings that a high-salt diet, or DOCA treatment that increases sodium in skin and muscle, increases contraction frequency while reducing contraction amplitude [19]. These observations are timely, since strategies to improve interstitial clearance currently target lymphatic network growth, although the efficacy appears to be context-dependent. Thus, activation of the VEGF-C–VEGFR-3 pathway to promote lymphangiogenesis can reduce kidney fibrosis and lessen cystic kidney disease in mice and rats [9]. Also, kidney-specific overexpression of VEGF-D before injury increased lymphatic density and amplified recovery from ischemia-reperfusion damage [28]. In contrast, inhibition of VEGFR-3 reduces kidney lymphangiogenesis, glomerulosclerosis, and tubulointerstitial fibrosis in a mouse model of diabetic kidney disease as well as fibrosis following UUO and ischemia-reperfusion [10]. Our data suggest that high interstitial sodium blunts lymphatic dynamics and may be a critical factor contributing to the efficacy of therapeutic intervention.

Currently, the first-line therapy to reduce sodium overload in a variety of diseases, including kidney disease, is inhibition of NKCC cotransporter with furosemide. Immunohistochemical staining clearly demonstrated NKCC1 in endothelial cells of renal collecting lymphatic vessels, and quantitation of mRNA showed increased gene expression in PAN vessels vs collecting vessels of uninjured kidneys (Figure 5). However, a high-sodium environment significantly reduced phosphorylation of NKCC1 in LECs, (Figure 6). Moreover, a high-sodium environment also reduced phosphorylation of SPAK, the upstream kinase of NKCC1, suggesting sodium dampens lymphatic contractility. Previous studies showed high salt downregulated phosphorylation and ubiquitination of WNK [29], which reduced expression of SPAK and NKCC1. Zeniya et al. showed suppressed phosphorylation of NKCC1 on mouse aortae fed a high-salt diet and stimulated phosphorylation of NKCC1 in mice on a low-salt diet [22]. Similar to our results with direct sodium exposure, a high-salt diet caused a divergent effect on the gene and protein expression of upstream kinases. Together, these data fit well with evidence that, aside from maintaining extracellular fluid volume, sodium acts as a signaling molecule.

NKCC1 activity can contribute to both vasoconstriction and vasodilation. Vasoconstrictors such as norepinephrine, endothelin, and angiotensin II directly activate NKCC1 activity in vascular smooth muscle cells, causing constriction, while NO and sodium nitroprusside inhibit NKCC1, resulting in vasodilation [30,31]. High-sodium environments reduce phosphorylated eNOS, which would predict reduced vasodilation but increased contractility. Indeed, inhibiting NO signaling with L-NAME decreased end diastolic and end systolic vessel diameter, the amplitude of contraction, calculated ejection fraction, and increased contraction frequency in renal lymphatic vessels (Figure 6B). Interestingly, previous studies confirm that a high-salt diet and/or direct exposure of lymphatic vessels to a high-sodium environment increases contraction frequency in skin and muscle lymphatics and inguinal lymphatic vessels of mice and rats [19,32,33]. 

Our data clearly show that a high-sodium environment directly blunts lymphatic dynamics (Figure 3). Since lymphatic vessels are exquisitely sensitive to environmental stimuli, other molecules within the renal interstitial compartment including vasoactive substances, for example, angiotensin II, may also play a role in lymphatic dynamic functions. However, comparison with vessels from PAN-injured kidneys exposed to a high-sodium environment revealed that renal injury is an additive contributor to lymphatic dysfunction (Figure 7). Thus, injured vessels exposed to high sodium showed diminution in their ability to respond to a pathological shift in their environment. This constellation of findings predicts impaired drainage of the renal interstitium in settings where a high interstitial sodium environment may prevail, such as in congestive heart failure, cirrhosis, and acute and chronic kidney disease. Moreover, these are the very conditions that show relative resistance to interventions that promote sodium excretion by inhibition of NKCC1. Notably, the ejection fraction in PAN-injured vessels is less affected by increasing concentrations of furosemide (Figure 9). Currently, therapeutic resistance to these agents centers on impaired delivery of the therapeutic to the relevant tubular segment. However, based on our data, we propose that dysfunction of renal lymphatic vessels is related to electrolyte abnormalities in the microenvironment of the kidney.

## 4. Materials and Methods

### 4.1. Animal Experiments

Male Sprague–Dawley rats (Charles River) weighing 180 g to 250 g were housed in a facility with 1:1 light/dark cycle. The animals were acclimated for at least 7 days and had free access to food and water. The well-established model of puromycin aminonucleoside nephropathy (PAN) was achieved by injecting puromycin aminoglycoside dissolved in 0.9% saline (125 mg/kg body weight i.p.). Rats injected with 0.9% saline served as controls. Eight days later, renal afferent lymphatic vessels were harvested for pressure myography. In a separate subset of PAN and control rats, renal lymph was collected using a glass pipette. The animal protocol was approved by Vanderbilt University Medical Center Institutional Animal Care and Use Committee in accordance with National Institutes of Health guidelines.

### 4.2. Magnetic Resonance Imaging Acquisition

Imaging experiments were performed in the Vanderbilt University Institute of Imaging Science (VUIIS) Center for Small Animal Imaging. Live animals were anesthetized and respiration and temperature were continuously monitored during imaging. Sodium MRI was acquired with custom-built, single-tuned sodium (^23^Na) surface coil (approximately 2 cm in diameter) placed over the kidney and the animal positioned prone in a 63 mm quadrature proton (1H) volume coil in a 9.4T scanner (Agilent Technologies, Santa Clara, CA, USA). Sodium standards (NaCl in milli-q water with concentrations 40, 70, 140 mmol/L) were incorporated in the image field-of-view (FOV) to calibrate ^23^Na signal intensity to standard sodium concentrations. Sodium MRI was acquired using a gradient echo multi-slice sequence with repetition time (TR) = 150 ms, echo time (TE) = 1.45 ms, FOV = 80 × 80 mm^2^, matrix = 32 × 32 interpolated to 128 × 128, slice thickness = 20 mm, and the number of experiments (NEX) = 100. Anatomical T1-weighted images were acquired in an identical FOV as sodium MRI with a fast spin-echo sequence (TR/TE = 2000/20 ms, matrix = 128 × 128, number of slices = 10, slice thickness = 2 mm, and NEX = 4 respiratory-triggered gated acquisitions), sufficient to locate renal compartments. 

Quantitative *T*_2_ mapping, which measures the transverse relaxation rate of water protons in tissue, is a commonly used technique both clinically and pre-clinically for identifying and evaluating edema [34,35]. Proton MRI for *T*_2_ relaxation time quantification was acquired at 7T (Bruker Avance III) in live animals with the kidneys centered in a 72 mm quadrature proton (1H) volume coil (Bruker Biospin). A water standard (3 mm NMR tube filled with 5 mM copper sulfate (CuSO4) in distilled water) was placed next to the left kidney. High spatial-resolution *T*_2_-weighted anatomical imaging was performed using a RARE (Rapid Acquisition with Relaxation Enhancement) sequence with TR/TE = 2000/45 ms, FOV = 70 × 70 mm^2^, slice thickness = 1 mm, matrix = 128 × 128, with 28–36 slices covering the kidneys bilaterally, and NEX = 8. In one axial slice through the center of each kidney, multi-spin-echo imaging was performed for *T*_2_-relaxation time mapping (TR = 2000 ms, echo spacing = 7 ms, 16 echoes, FOV = 70 × 70 mm^2^, matrix = 64 × 64, NEX = 4).

#### Image Analysis

Renal tissue sodium content maps were calculated voxel-wise. First, a calibration curve was calculated as the least-squares linear regression fit of the mean ^23^ Na signal intensity in each standard solution to their known concentrations (40, 70, 140 mmol/L). The calibration curve was applied voxel-wise to calculate tissue sodium content (TSC, mmol/L) in the imaged kidney. *T*_2_ relaxation time maps were calculated voxel-wise for each kidney using a nonlinear least-squares fit of the 1H signal intensity at each echo time, normalized by 1H signal in a standard water phantom, to a monoexponential decay function. Anatomical images were used to segment regions of interest (ROIs) manually in renal compartments consisting of the cortex, medulla, and papilla. Mean TSC (mmol/L) and *T*_2_ relaxation time (ms) metrics were calculated in each ROI and preserved for statistical analyses. 

### 4.3. Serum and Lymph Sodium Analysis

Colorimetric sodium assay kit (Abcam) was used to measure sodium concentration in serum and lymph according to the manufacturers’ instructions.

### 4.4. Measurement of Lymphatic Vessels Contractility

Afferent extra-renal lymphatic collecting vessels were isolated by microdissection and lymphangions mounted on glass pipets in vessel perfusion chambers as reported [17]. A digital image capture system (IonOptix) was used to record pre-valve intraluminal diameters. Vessels were warmed to 37 °C, pressurized to 0.5 mmHg using a column of Krebs buffer, and allowed to equilibrate (20–60 min) before incrementally increasing the intraluminal pressure to 2.5 mmHg. Vessels that failed to contract spontaneously were excluded from further study. For high-sodium environment studies, the vessels were exposed to a modified high-sodium Krebs buffer (see below). Some vessels were also challenged with increasing concentrations of furosemide (10-7-10-3M, Hospira). For each experimental condition, lumen diameters were allowed to plateau (20–40 min) before moving to the next condition. Single vessels were exposed to 1 to 3 compounds over the course of each experiment. We found no difference in response or viability based on the order of compound administration. As previously reported [17,36], the amplitude of contraction was measured as the difference between the end diastolic diameter and end systolic diameter (EDD-ESD). The ejection fraction was calculated as (EDD2-ESD2)/EDD2.

#### Buffers

Standard Krebs buffer contained the following: 109 mM NaCl, 4.7 mM KCl, 2.5 mM CaCl_2_, 0.9 mM MgSO_4_, 1 mM KH_2_PO4, 11.1 mM glucose, 34 mM NaHCO_3_. High-sodium Krebs buffer contained the following: 151 mM NaCl, 4.7 mM KCl, 2.5 mM CaCl_2_, 0.9 mM MgSO_4_, 1 mM KH_2_PO_4_, 11.1 mM glucose, 34 mM NaHCO_3_.

### 4.5. Reverse Transcription Quantitative Real-Time PCR

Lymphatic vessels were homogenized in RLT and β-ME buffer using a rotating homogenizer. Cultured cells were harvested directly from culture plates with RLT-β-ME buffer and the RNA extracted using Qiagen RNeasy Mini Kit by standard protocol. TaqMan Reverse Transcription Kit (Applied Biosystems, Waltham, MA, USA) was used for reverse transcription. β-actin was used as endogenous control and fold difference in gene expression data was calculated by 2^−^^△△Ct^ method. Human and rat NKCC1 and β-actin primers were bought from Thermo Fisher Scientific. 

### 4.6. Immunohistochemical Staining

Renal lymphatic vessels were collected from rats, fixed in the 4% paraformaldehyde, and embedded in paraffin. Three-micron paraffin sections were stained with the standard protocol. For NKCC1 staining, tissues were deparaffinized and then antigen retrieved with citrate buffer (pH = 6.0). After blocking with 2.5% normal horse serum, the tissue was incubated with anti-NKCC1 antibody (Boster Bio, 1:1000, Pleasanton, CA, USA) overnight and then incubated with anti-rabbit secondary antibody. Negative control was prepared by omitting the primary antibody.

### 4.7. Cell Culture

Adult lymphatic endothelial cells (LECs) (PromoCell) were cultured with endothelial cell growth medium (PromoCell). After starvation with serum-free medium at passage 5–6, LECs were incubated in normal medium (control group, (Na+) = 145 mmol/L) and high-sodium medium ((Na+) = 185 mmol/L) for 24 h. Urea (Sigma–Aldrich, St. Louis, MO, USA) group was used to control for potential osmolarity effects. RT-PCR was performed for *Nkcc1* mRNA quantification. Total protein was extracted for quantitation of NKCC1, SPAK, and eNOS.

### 4.8. Western Blot

Cells were lysed in RIPA with phosphatase inhibitor and protease inhibitor (Roche). Anti-phospho-NKCC1 antibody (Millipore, 1:5000), anti-phospho-SPAK antibody (Millipore, 1:4000), anti-phospho-eNOS antibody (Millipore, 1:8000), and anti-rabbit secondary antibody were used to detect phosphorate-NKCC1 and phosphorate-SPAK. The two most thoroughly studied sites of phospho-eNOS are the activation site Ser1177 and the inhibitory site Thr495. Several protein kinases, including Akt/PKB, PKA, and AMPK activate eNOS by phosphorylating Ser1177 in response to various stimuli. In our study, we used anti-phospho-eNOS antibody (Ser1177). Sample loading was measured by β-actin (1:30,000) with anti-mouse as secondary antibody. 

### 4.9. Statistical Analysis

Data are presented as mean ± standard error of mean. *T*-test was used for comparison between the two groups, and the ANOVA analysis was used for the comparison between multiple groups. *p* < 0.05 was considered to be statistically significant.

## 5. Conclusions

Based on our data, we propose that dysfunction of renal lymphatic vessels is related to electrolyte abnormalities. Furthermore, although lymphangiogenesis has been firmly established to accompany these conditions, our data suggest that sodium-induced lymphatic dysfunction compounds the problem of impaired fluid clearance in the setting of kidney injury. Sodium accumulation suppresses the pumping function of renal lymphatic vessels by inhibiting the SPAK-NKCC1 cascade. These results imply that the lymphatic system should be viewed as a potential target in disease characterized by sodium accumulation, such as various renal diseases or heart failure.

## Data Availability

All data and other materials can be obtained from authors.
